# Supplementary material for: Low Alanine Aminotransferase Cut-Off for Predicting Liver Outcomes; A Nationwide Population-Based Longitudinal Cohort Study
Source: J Clin Med. 2019 Sep 11;8(9):1445. doi: 10.3390/jcm8091445 (PMC6780691; doi:10.3390/jcm8091445)
Supplement: Supplementary file 1 [file jcm-08-01445-s001.pdf]

Supplement table 1. Survival table of unfavorable liver related outcomes according to low ALT cut-off

Patients at risk			Time (years)					
			0	2.5	5	7.5	10	11
Male	ALT ≤ 40	At risk	160462	160462	158683	155873	152515	93609
		Liver-related mortality		40	38	32	38	4
		At risk	160629	160629	157821	154290	150056	106912
		Hepatocellular carcinoma		2808	3531	4234	4489	532
		At risk	160582	160582	158592	155859	152555	109489
		Decompensated events		120	156	97	103	7
	ALT > 40	At risk	24824	24824	24603	24170	23693	17247
		Liver-related mortality		15	38	30	43	7
		At risk	24881	24881	24291	23632	22904	16442
		Hepatocellular carcinoma		590	659	728	828	87
		At risk	24878	24878	24579	24142	23645	13997
		Decompensated events		60	83	58	67	7
Female	ALT ≤ 30	At risk	144582	144582	144906	143563	141538	100705
		Liver-related mortality		9	10	17	11	1
		At risk	145763	145763	144374	142381	139880	101781
		Hepatocellular carcinoma		1390	1993	2501	2895	369
		At risk	145744	145744	144947	143495	141758	101672
		Decompensated events		52	67	64	47	5
	ALT > 30	At risk	6925	6925	6879	6764	6662	3228
		Liver-related mortality		4	11	11	1	0
		At risk	6942	6942	6802	6626	6435	4542
		Hepatocellular carcinoma		140	176	191	197	25
		At risk	6935	6935	6864	6769	6660	3229
		Decompensated events		9	18	14	13	1

ALT: alanine transaminase

Supplement table 2. Survival table of unfavorable liver related outcomes according to conventional ALT cut-off

Patients at risk			Time (years)					
			0	2.5	5	7.5	10	11
Male	ALT ≤ 30	At risk	130877	130877	129335	127018	124199	76188
		Liver-related mortality		31	23	168	25	4
		At risk	131022	131022	128733	125822	122338	86966
		Hepatocellular carcinoma		2289	2911	3484	3728	444
		At risk	130987	130987	129315	126984	124240	88964
		Decompensated events		81	117	67	79	5
	ALT > 30	At risk	54377	54377	53891	53019	52022	37794
		Liver-related mortality		24	53	38	56	7
		At risk	54488	54488	53379	52100	50622	36358
		Hepatocellular carcinoma		1109	1279	1478	1589	175
		At risk	54480	54480	53853	52987	51963	31741
		Decompensated events		99	122	87	92	9
Female	ALT ≤ 19	At risk	89836	89836	89358	88700	87331	62231
		Liver-related mortality		4	4	5	3	1
		At risk	90048	90048	89232	88072	86591	62998
		Hepatocellular carcinoma		816	1160	1481	1683	209
		At risk	90026	90026	89541	88613	87555	25875
		Decompensated events		32	32	28	23	1
	ALT > 19	At risk	62563	62563	62265	61627	60748	55061
		Liver-related mortality		9	17	23	9	0
		At risk	62657	62657	61944	60935	59724	43442
		Hepatocellular carcinoma		714	1009	1211	1409	185
		At risk	62648	62648	62284	61625	60806	43461
		Decompensated events		29	53	50	37	5

ALT: alanine transaminase
